# Identification of an Ortholog of MALT1 from Shrimp That Induces NF-κB-Mediated Antiviral Immunity

**DOI:** 10.3390/v15122361

**Published:** 2023-11-30

**Authors:** Haiyang Wang, Bang Xiao, Shihan Chen, Jianguo He, Chaozheng Li

**Affiliations:** 1 State Key Laboratory of Biocontrol, Southern Marine Science and Engineering Guangdong Laboratory (Zhuhai), School of Marine Sciences, Sun Yat-sen University, Guangzhou 510275, China; 2 Guangdong Provincial Key Laboratory of Marine Resources and Coastal Engineering/Guangdong Provincial Key Laboratory for Aquatic Economic Animals, School of Life Sciences, Sun Yat-sen University, Guangzhou 510275, China; 3 Maoming Branch, Guangdong Laboratory for Lingnan Modern Agriculture, Maoming 525000, China; 4 China-ASEAN Belt and Road Joint Laboratory on Marine Aquaculture Technology, Guangzhou 510275, China

**Keywords:** MALT1, *Litopenaeus vannamei*, WSSV, AMPs, NF-κB

## Abstract

MALT1 (mucosa-associated lymphoid tissue lymphoma translocation protein 1) serves as a pivotal mediator for NF-κB activation in response to a wide spectrum of transmembrane receptor stimuli. In the present study, a homolog of MALT1, named LvMALT1, is cloned from the Pacific white shrimp (*Litopenaeus vannamei*) and its potential function in shrimp innate immunity is explored. The open reading frame of LvMALT1 is 2364 bp that encodes 787 amino acids. The predicted LvMALT1 protein structure comprises a death domain, three immunoglobulin domains, and a caspase-like domain, exhibiting remarkable similarity to other homologs. LvMALT1 is a cytoplasmic-localized protein and could interact with LvTRAF6. Overexpression of LvMALT1 induces the activation of promoter elements governing the expression of several key antimicrobial peptides (AMPs), including penaeidins (PENs) and crustins (CRUs). Conversely, silencing of LvMALT1 leads to a reduction in the phosphorylation levels of Dorsal and Relish, along with a concomitant decline in the in vivo expression levels of multiple AMPs. Furthermore, LvMALT1 is prominently upregulated in response to a challenge by the white spot syndrome virus (WSSV), facilitating the NF-κB-mediated expression of AMPs as a defense against viral infection. Taken together, we identified a MALT1 homolog from the shrimp *L. vannamei*, which plays a positive role in the TRAF6/NF-κB/AMPs axis-mediated innate immunity.

## 1. Introduction

MALT1, or mucosa-associated lymphoid tissue lymphoma translocation protein 1, is a pivotal adaptor protein central to the regulation of the NF-κB pathway. It acts as a scaffold protein, facilitating the initiation of downstream signaling pathways [1]. In the context of organismal immunity against viral pathogens, a complex interaction of various cells and signaling pathways comes into play. MALT1 emerges as a key orchestrator of these intricate processes [2,3]. Structurally, MALT1 comprises distinct domains, including a death domain (DD) at the N-terminus, two immunoglobulin (Ig)-like domains, a caspase-like domain, an additional Ig-like domain, and an unstructured C-terminal region [4]. The DD regions in the N-terminus directly bind to B-cell lymphoma 10 (BCL10), an interaction critical for the formation of the oligomeric CARMA1–BCL10–MALT1 (CBM) complex. This complex, in turn, triggers the downstream activation of the nuclear factor-κB (NF-κB) signaling pathway. The two Ig-like domains following the DD region play a supportive role in stabilizing the MALT1–BCL10 binding [4,5]. Furthermore, multiple binding sites for tumor necrosis factor receptor-associated factor 6 (TRAF6) have been identified within the caspase-like domain and the C-terminal region of MALT1 [6]. TRAF6, functioning as an E3 ubiquitination ligase, ubiquitinates various substrates, including MALT1, which features several C-terminal lysine (K) residues. This ubiquitination process is instrumental in the recruitment of IKKγ and the induction of NF-κB activation [7,8]. While MALT1 has been extensively studied in vertebrates, the identification of MALT1 homologs in invertebrates remains limited.

Shrimp aquaculture serves as a crucial source of animal protein for human consumption, addressing the ever-growing global demand for protein-rich food. *Litopenaeus vannamei*, a prominent shrimp species, holds significant importance in the aquaculture industry worldwide. However, as with all farmed species, *L. vannamei* is vulnerable to a spectrum of diseases that engender substantial economic losses [9,10]. White spot syndrome (WSS), attributed to the white spot syndrome virus (WSSV), stands out as the most pernicious viral pathogen afflicting shrimp, resulting in 100% mortality within only 10 days of infection. It has thus emerged as a formidable impediment to cultured shrimp production [11]. Over the past decades, shrimp innate immunity has garnered considerable attention due to the devastating impact of diseases. Numerous immune-related proteins and pathways in shrimp have been identified, including the NF-κB pathway, which plays a pivotal role during WSSV infection [12,13,14]. Shrimp exhibit two NF-κB pathways, the Toll and immune deficiency (IMD) pathways. Dorsal serves as the principal transcription factor in the Toll signaling pathway [15], while Relish assumes a critical role in the IMD signaling pathway [16]. These pathways are integral in regulating the expression of antimicrobial peptides (AMPs), such as the penaeidin (PENs) and crustin (CRUs) families, which have demonstrated efficacy against WSSV [17,18,19,20]. Nevertheless, the involvement of MALT1 in NF-κB pathway regulation and its potential role in combating WSSV infection remain unclear.

In this study, we cloned and identified a MALT1 homolog, named LvMALT1, from the shrimp *L. vannamei*, which exhibits a remarkable evolutionary conservation in the MALT1 domain. Notably, LvMALT1 plays a role in antiviral defense, potentially through its interaction with LvTRAF6 to regulate the NF-κB-AMPs-mediated antiviral routes. These findings not only shed light on the regulation of signals mediated by MALT1 in invertebrates but also offer valuable insights for disease control and resistant breeding in the realm of shrimp aquaculture.

## 2. Methods and Materials

### 2.1. Plasmid Constructions

The open reading frame (ORF) of *LvMALT1* (2361 bp without a termination codon) was cloned into pAc5.1-HA (Warner Bio, Wuhan, China) and pAc5.1-RFP (Warner Bio, Wuhan, China) vectors to generate pAc-LvMALT1-HA and pAc-LvMALT1-RFP plasmids. Three truncated forms of *LvMALT1*, including the regions of 1–100 aa, 101–300 aa and 301–787 aa, were cloned into pAc5.1-HA to generate different protein region expression plasmids. Reporter gene plasmids containing the promoters of *Drosophila* antimicrobial peptide genes, including attacin A (Atta), metchnikowin (Mtk), cecropin A (CecA), drosomycin (Drs), diptericin (Dipt), defensin (Def), and *L. vannamei* antimicrobial peptide genes, such as PENs (PEN2, PEN3, PEN4) and CRUs (CRU1, CRU2, CRU3), along with an NF-κB activating element luciferase reporter plasmid, were obtained from our previous studies [21,22]. The plasmid pAc–LvTRAF6–GFP was previously developed in our laboratory [23]. Primer sequences are listed in Table 1.

### 2.2. Sequence and Phylogenetic Analysis of LvMALT1

The protein domains in LvMALT1 were predicted by using the SMART program (http://smart.embl-heidelberg.de/, accessed on 10 October 2022). Protein sequences of MALT1 homologs were retrieved from the NCBI database by BLAST. Sequence alignments of MALT1 homologs were analyzed using the ClustalX v2.0 program [24] and then visualized by using GeneDoc. The phylogenetic tree was constructed based on the full-length amino acid sequences of MALT1 proteins by utilizing MEGA 5.0 software with the neighbor-joining (NJ) method [25].

### 2.3. Tissue Expression and WSSV Challenge Analysis by Quantitative RT-PCR

Shrimp (*L. vannamei*, ~5 g weight each) were purchased from Guangdong Hisenor Group Co., Ltd., Guangzhou, P. R. China. Pathogens including WSSV, IHHNV, DIV1, SHIV, TSV, YHV, EHP, and VP_AHPND_ were detected in shrimp before conducting experiments to ensure that the shrimp were pathogen free by using the following methods [26,27,28,29,30]. Shrimp were cultured in aerated seawater for three days and fed with commercial food (Hisenor, Guangzhou, China) three times a day before the experiments. For tissue expression analysis, eight types of tissues including hemocyte, nerve, hepatopancreas, intestine, stomach, heart, antennae, and gill were sampled. Gills of WSSV challenged shrimp were collected at 0, 4, 8, 12, 24, 36, 48, and 72 h post injection (hpi), and each sample was collected and pooled from 10 shrimp. The experiments were performed on three biological replicates. Total RNA was isolated by using Trizol reagent (Life Technologies, Gaithersburg, MD, USA), and dissolved in Nuclease-free Water (Takara, Dalian, China). The concentration and quality of RNA was detected by using an ultraviolet spectrophotometer NANO 2000 (Thermo, Waltham, MA, USA) and all of the RNA had an OD260/OD280 ratio of ~2.0. Total RNA (1 µg) was used in 20 µL of a reverse transcription reaction by using TransScripOne-Step gDNA Removal and cDNA Synthesis SuperMix for the PCR kit (TransGenBiotech, Guangzhou, China) for the synthesis of the first-strand cDNA. Expression levels of Lv*MALT1* were determined by quantitative PCR using TB Green Fast qPCR Mix (Takara, Dalian, China) and calculated using the Livak (2^−ΔΔCT^) method [31] after normalization to *L. vannamei* EF-1α. Primer sequences are listed in Table 1. 

### 2.4. Co-Immunoprecipitation

To investigate the interaction between LvMALT1 and LvTRAF6, HA-tagged LvMALT1 was co-transfected with LvTRAF6-GFP or GFP (as a control) into S2 cells. Additionally, LvTRAF6-GFP was co-transfected with three truncated forms of LvMALT1 (1–100 aa, 101–300 aa, 301–787 aa), each tagged with HA. After 48 h of plasmid transfection, cells were collected and lysed using IP Lysis Buffer (Pierce; cat. no. 87788) supplemented with a Halt Protease Inhibitor Cocktail (Thermo, Waltham, MA, USA, 87788). Approximately 90% of the cell lysate was incubated with agarose affinity gel containing anti-GFP (MBL International Corporation, Woburn, MA, USA, D153-8). The remaining 10% of the cell lysate was used as input for reference. All samples underwent SDS-PAGE assays. For Western blotting, we used rabbit anti-GFP antibody (Sigma-Aldrich, St.Louis, MO, USA, SAB4301138) as the primary antibody, and anti-rabbit IgG HRP-conjugate (Promega, Madison, WI, USA, W401B) as the secondary antibody. All antibodies were diluted in TBS.

### 2.5. Confocal Laser Scanning Microscopy

*Drosophila* S2 cells were cultured in a 12-well plate, and each well was transfected with 0.5 μg pAc–LvMALT1–RFP and pAc–LvTRAF6–GFP following a previously described method [22]. Subcellular localization analyses were conducted using a Hoechst 33258 (Beyotime, Shanghai, China, C1011) and were visualized with a confocal microscope (Leica, Wetzlar, Germany, TCSSP8).

### 2.6. Dual-Luciferase Reporter Assay

*Drosophila* S2 cells were cultured in a 96-well plate, and each well was transfected with 0.05 μg firefly luciferase reporter gene plasmids, 0.01 μg pRL-TK renilla luciferase plasmids, and 0.05 μg expression plasmids (the full-length or truncation forms of LvMALT1 or pAc5.1A-HA). The expression levels of those reporter genes were measured by using a Dual-Glo Luciferase Assay System kit (Promega; cat. no. E2920) according to the manufacturer’s instructions. All experiments were performed on three biological replicates.

### 2.7. Knockdown of LvMALT1 Expression by dsRNA-Mediated RNA Interference

The dsRNA constructs, dsRNA-LvMALT1 and dsRNA-GFP, were generated using primers (Table 1) containing a 5′ T7 RNA polymerase binding site and synthesized through in vitro transcription using a T7 RiboMAX Express RNAi System kit (Promega, Madison, WA, USA). In the experimental groups, each shrimp received an intra-muscular injection of LvMALT1 dsRNA (10 µg dsRNA in 50 µL PBS), while the control groups were injected with an equivalent amount of GFP dsRNA. At 48 h post dsRNA injection, gill samples were collected from each group to assess knockdown efficiency (primer information is provided in Table 1), and the expression levels of PENs and CRUs were determined via quantitative PCR (qPCR). The experiments were performed on three biological replicates. Hemocytes from the shrimp were harvested for Western blotting to measure the phosphorylation levels of LvDorsal and LvRelish. The primary antibodies used in the Western blotting analysis included rabbit anti-Phospho-LvRelish antibody (Genecreate, Wuhan, China) for detecting LvRelish phosphorylation [32], anti-NF-κB p65 (phospho S276) antibody (Abcam, Cambridge, UK, ab194726) for detecting LvDorsal phosphorylation [33], and mouse anti-actin antibody (Merck Millipore, Billerica, MA, USA, MAB1501) for use as an internal control. For the secondary antibody, anti-rabbit IgG HRP-conjugate (Promega, Madison, WI, USA, W401B) was employed. Primer sequences are listed in Table 1.

### 2.8. WSSV Challenge Experiments in LvMALT1 Knocked Down Shrimp

Disease-free shrimp, with a sample size of 60 in each group, were administered a 50 μL solution of dsRNA (either LvMALT1 dsRNA or GFP dsRNA, both diluted in PBS) at a dosage of 10 μg of dsRNA per shrimp. Forty-eight hours after the initial dsRNA injection, half of the shrimp in each group received an additional injection. This second injection included approximately 1 × 10^5^ copies of WSSV particles, which were diluted in 50 μL of PBS. The remaining shrimp in each group were injected with 50 μL of PBS alone. Following these injections, the shrimp were reared for 7 days in tanks. Mortality rates in each group were recorded every 4 h, and the differences between the groups were subsequently analyzed using the Mantel–Cox (log-rank χ^2^ test) method in GraphPad Prism 9 software.

Another experiment was conducted to monitor WSSV replication and the expression of antimicrobial peptides (AMPs) in LvMALT1-silenced shrimp (*n* = 30 each group). Gill tissues were collected from 12 individual shrimp in the dsRNA-LvMALT1+WSSV and dsRNA-GFP+WSSV groups at 48 h post-infection to extract DNA. The quantities of WSSV genome copies were determined through absolute quantitative PCR using the primers WSSV32678–F/WSSV32753-R and a TaqMan fluorogenic probe, as previously described [34]. Subsequently, the WSSV genome copy numbers in 0.1 μg of shrimp gill DNA were calculated. At 48 h post-WSSV infection, three hemocyte samples were collected, each consisting of three shrimp. qPCR was then performed to assess the expression of LvMALT1 and AMPs in WSSV-infected shrimp. All experiments were performed on three biological replicates. Primer sequences are listed in Table 1.

### 2.9. Ethics Statement

All animal experiments were approved by the Institutional Animal Care and Use Committee (IACUC) of Sun Yat-Sen University (Approval No. SYSU-IACUC-2023-B0005, 18 January 2023). 

## 3. Results

### 3.1. Sequence Analysis and Phylogenetic Tree of LvMALT1

**Figure 1:**
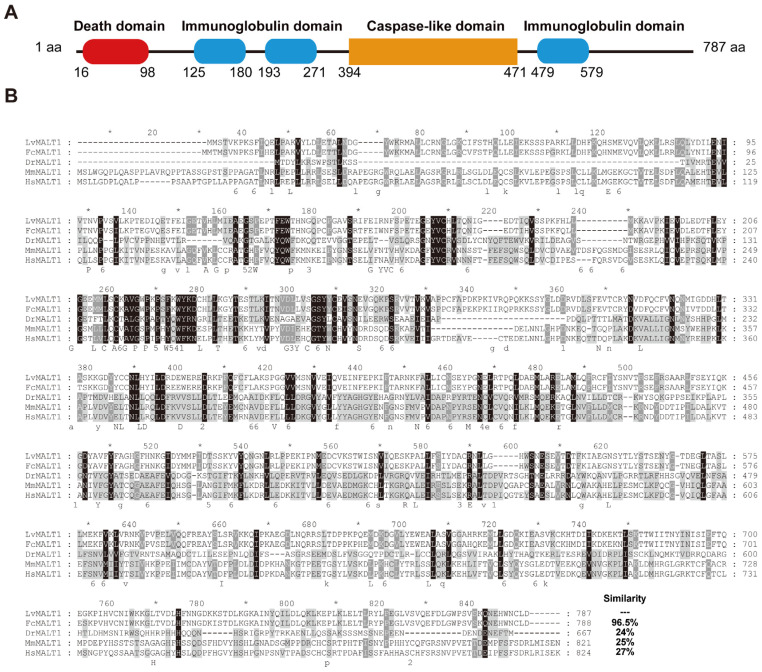
Sequence analysis of LvMALT1. (**A**) Architecture and location representation of the characteristic domains of LvMALT1. (**B**) Multiple sequence alignment of MALT1 proteins. The identical amino acid residues are shaded in black, while the similar residues are shaded in gray.

Based on both transcriptome and genome data sources [35,36], the *LvMALT1* transcript from *L. vannamei* revealed that the *LvMALT1* open reading frame (ORF) spans 2364 base pairs, encoding a 787-amino acid protein, with a calculated molecular weight of 90.2 kDa. The LvMALT1 protein exhibits structural features, including a death domain in the N-terminal region (amino acids 16–98), three immunoglobulin-like domains (amino acids 125–180, 193–271, 479–579), and a caspase-like domain in the C-terminal region (amino acids 394–471) (Figure 1A).

The analysis of multiple sequence alignments indicated that the full-length LvMALT1 protein shares a similarity range of 24% to 96.5% with other MALT1 proteins (Figure 1B). The highest level of homology (96.5%) was observed between LvMALT1 and FcMALT1 (*Fenneropenaeus chinensis*), suggesting that MALT1 genes are conserved across a wide spectrum of species, extending from invertebrates to mammals. These findings collectively support the classification of LvMALT1 as a member of the MALT1 protein family.

**Figure 2:**
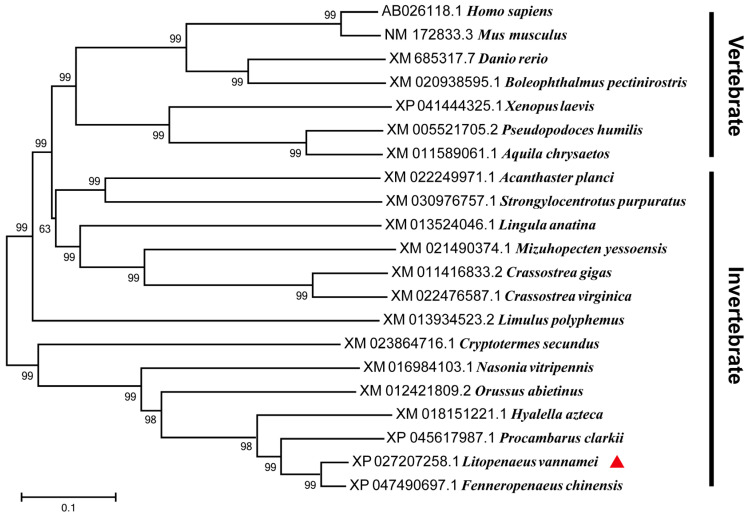
Phylogenetic tree analysis of the full-length amino acid sequences of MALT1 proteins from various species (LvMALT1 marked by a red triangle). Sequence alignment was performed by using ClustalW and the tree was constructed by using the NJ method in MEGA5.0 software. GenBank accession number of each MALT1 is shown before their scientific names.

The phylogenetic tree was constructed using the neighbor-joining (NJ) method, based on the full-length sequences of MALT1 homologs. LvMALT1 and its homologs were categorized into two distinct branches, vertebrates and invertebrates. LvMALT1 formed a cluster with MALT1 homologs from other crustaceans, placing it within the invertebrate branch of the phylogenetic tree alongside arthropods and mollusks (Figure 2).

### 3.2. Expression of Immune Challenged Shrimp

**Figure 3:**
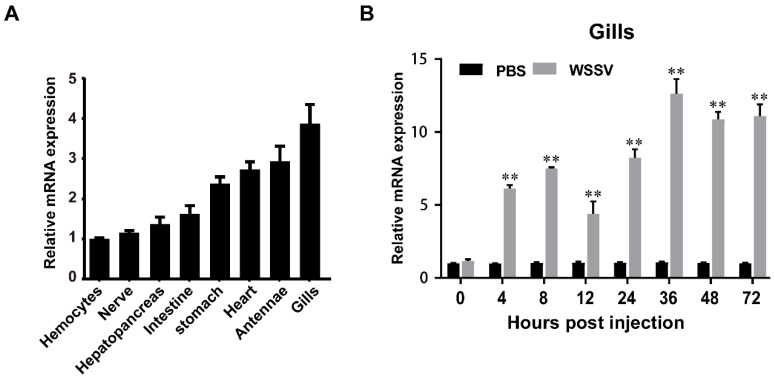
Expression levels of *LvMALT1* in uninfected and immune challenged shrimp. (**A**) Transcription levels of *LvMALT1* in different tissues were determined by qPCR. Expression level in hemocytes is set to 1.0. (**B**) Expression profiles of *LvMALT1* in gills from PBS and WSSV challenged shrimp. Expression level at each time point was normalized to 0 h post PBS injected group (** *p* < 0.01). Data are presented as means ± SD of triplicate assays. Experiments were performed on three biological replicates.

To determine the tissue distribution of *LvMALT1*, we conducted quantitative RT-PCR analysis on eight different tissues obtained from healthy shrimp. Our results revealed that *LvMALT1* transcripts were discernible in all the tissues examined. The highest mRNA expression of *LvMALT1* was observed in the gills, exhibiting approximately a 3.7-fold increase compared to hemocytes, that were set as the reference (1.0). Additionally, substantial expression levels were detected in the antennae (approximately 2.9-fold), heart (about 2.7-fold), and stomach (roughly 2.4-fold), while other examined tissues exhibited comparatively lower expression levels (Figure 3A). In light of our study’s primary focus on *LvMALT1*’s immune function, we selected the gills for further investigation. Within the gills of WSSV-infected shrimp, *LvMALT1* expression consistently exhibited an upward trend, with a prominent peak (~12.6-fold increase) observed at the 36-h mark (Figure 3B).

### 3.3. Interactions between LvMALT1 and LvTRAF6

**Figure 4:**
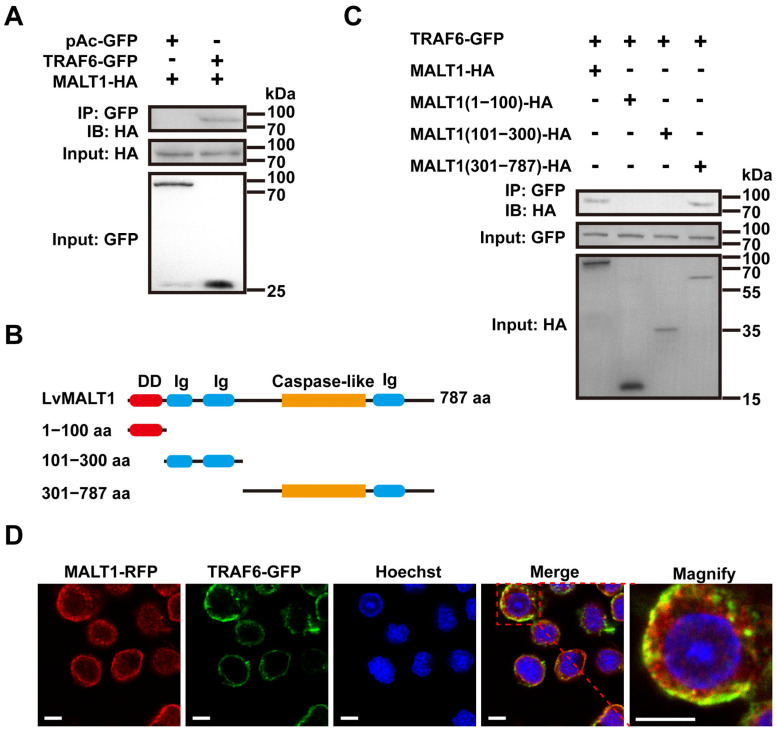
LvMALT1 interacted with LvTRAF6. (**A**) HA-tagged LvMALT1 was coprecipitated with GFP-tagged LvTRAF6, but not the control GFP protein; (**B**) Schematic representation of the full-length and truncation forms (1–100 aa, 101–300 aa, 301–787 aa) of LvMALT1; (**C**) HA-tagged LvMALT1 (301–787 aa) could interact with GFP-tagged LvTRAF6. (**D**) Subcellular localization of LvMALT1 and LvTRAF6. Scale bar: 5 μm.

MALT1 assumes a pivotal role in the signaling cascade bridging antigen receptors and the transcription factor NF-κB [37,38]. This function is executed by MALT1 binding to TRAF6, an event that triggers TRAF6 oligomerization and, subsequently, activates TRAF6’s ligase activity. This activation leads to the polyubiquitination of IKKs, culminating in the activation of NF-κB within T lymphocytes [6]. To examine the interaction between LvMALT1 and LvTRAF6, we conducted Co-IP assays employing the S2 cell line. As illustrated in Figure 4A, LvMALT1-HA exhibited an interaction with LvTRAF6-GFP but not with the GFP-tagged control. In a quest to understand more about the interaction between LvMALT1 and LvTRAF6, we generated three truncated forms of LvMALT1, namely LvMALT1 (1–100 aa), LvMALT1 (101–300 aa), and LvMALT1 (301–787 aa) (Figure 4B). These truncated forms of LvMALT1-HA were co-transfected with LvTRAF6-GFP. The results, as shown in Figure 4C, revealed that LvMALT1 (301–787 aa) displayed an interaction with LvTRAF6-GFP, mirroring findings in mammals. Consolidating our results, we carried out subcellular colocalization analysis of LvMALT1 and LvTRAF6, wherein LvMALT1-RFP and GFP-tagged LvTRAF6 predominantly colocalized as yellow fluorescence within the cytoplasm (Figure 4D).

### 3.4. LvMALT1 Induces NF-κb-Mediated Antimicrobial Peptides Expression

**Figure 5:**
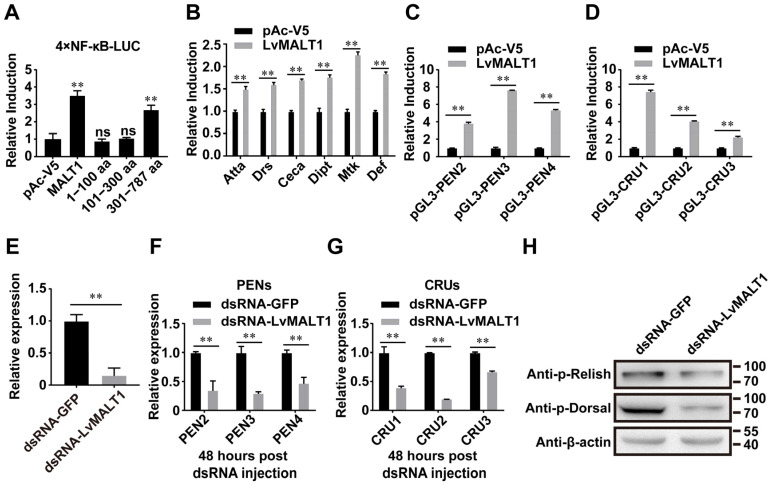
LvMALT1 induces NF-κB-mediated antimicrobial peptide expression. (**A**) Effects of LvMALT1 on the activity of *L. vannamei* NF-κB. The over-expression of LvMALT1 (full-length) and LvMALT1 (301–787 aa), but not LvMALT1 (1–100 aa) and LvMALT1 (301–787 aa), induced artificial promoters that contained NF-κB binding motifs. (**B**) Over-expression of LvMALT1 (full-length) could induce promoter activities of *Drosophila* AMPs (Atta, Drs, Ceca, Dipt, Mtk, Def) in S2 cells. (**C**,**D**) Over-expression of LvMALT1 (full-length) could induce promoter activities of shrimp PENs (**C**) and CRUs (**D**) in S2 cells. Bars (**A**–**D**) indicate mean ± SD of luciferase activities (*n* = 6). (**E**) Knockdown efficiency of LvMALT1 was confirmed by quantitative RT-PCR. (**F**,**G**) Expression of PENs (**F**) and CRUs (**G**) in LvMALT1 knocked-down shrimp. (**H**) Phosphorylation level of LvRelish and LvDorsal in dsRNA-GFP and dsRNA-LvMALT1-treated shrimp. Bars indicate mean ± SD of three samples and statistical significance was calculated by Student’s *t*-test (** *p* < 0.01, ns, no significance). Experiments were performed on three biological replicates.

To further investigate the mechanisms underpinning LvMALT1’s role in the NF-κB pathway of *L. vannamei*, we conducted an NF-κB reporter gene assay using LvMALT1 and its three truncated forms. As depicted in Figure 5A, the overexpression of LvMALT1 and LvMALT1 (301–787 aa) led to a significant up-regulation of NF-κB pathway activity, resulting in approximately 2.8-fold and 2.4-fold increases compared to the control. LvMALT1 also exhibited the capacity to enhance the expression of various antimicrobial peptides (Atta, Drs, Ceca, Dipt, Mtk, Def) from *D. melanogaster*, albeit to varying degrees (Figure 5B). Given that PENs and CRUs are under the regulatory control of the NF-κB pathway and contribute to shrimp’s antiviral and antibacterial immunity [17,18]. We proceeded to explore the impact of LvMALT1 on the promoter activities of PENs and CRUs. Our findings demonstrated that LvMALT1 could indeed elevate the transcription levels of these crucial shrimp AMPs (Figure 5C,D). To summarize, these results collectively affirm the in vitro activation of NF-κB pathways by LvMALT1. To bolster our evidence, we executed RNA interference (RNAi) experiments to investigate the relationship between LvMALT1 and the NF-κB pathway in vivo. We designed and synthesized dsRNA-LvMALT1, specifically targeting LvMALT1 transcription, and assessed the efficacy of LvMALT1 silencing 48 h post-dsRNA injection. The control groups received dsRNA-GFP. Notably, the dsRNA-LvMALT1 groups exhibited a significant reduction, reaching approximately 0.15-fold of the control group, a level sufficient for subsequent experiments (Figure 5E). Moreover, the transcription levels of LvPEN2, LvPEN3, LvPEN4, LvCRU1, LvCRU2, and LvCRU3 experienced substantial down-regulation to about 0.35-fold, 0.29-fold, 0.47-fold, 0.39-fold, 0.20-fold, and 0.67-fold, respectively, when compared to those of the dsRNA-GFP group at 48 h post-dsRNA injection (Figure 5F,G), consistent with the in vitro results (Figure 5C,D). Furthermore, we conducted in vivo examinations to explore the potential impact of LvMALT1 on the phosphorylation of LvDorsal and LvRelish through RNAi experiments. The phosphorylation levels of LvDorsal and LvRelish demonstrated a decrease following MALT1 inhibition (Figure 5H). In conclusion, these findings collectively attest to the role of LvMALT1 in NF-κB activation and its ability to induce the expression of PENs and CRUs.

### 3.5. Function of LvMALT1 during WSSV Infection

**Figure 6:**
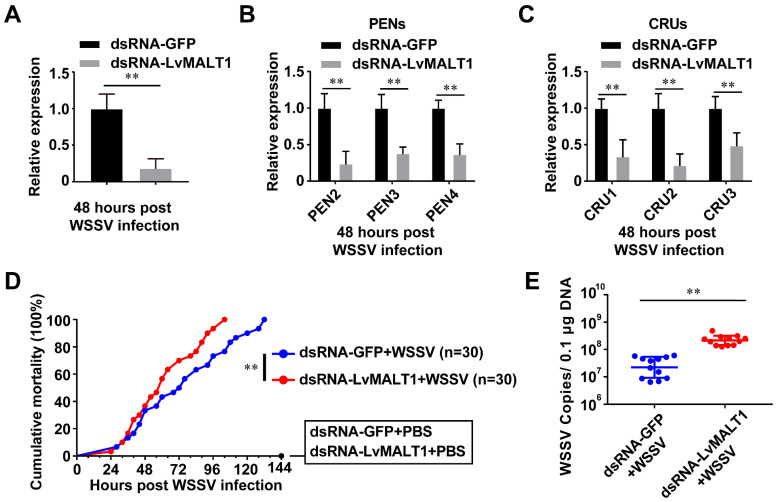
Function of LvMALT1 during WSSV infection. (**A**–**C**) Relative expression of LvMALT1 (**A**), PENs (**B**), and CRUs (**C**) in LvMALT1 knocked-down shrimp at 48 h post WSSV infection. (**D**) Cumulative mortality of LvMALT1 knocked-down shrimp after WSSV infection. (**E**) WSSV genome copies in gill tissues. Bars indicate mean ± SD of three samples and statistical significance calculated by Student’s *t*-test (** *p* < 0.01). Experiments were performed on three biological replicates.

The pivotal role of the shrimp NF-κB pathway in the antiviral immune response has been documented [13]. To assess LvMALT1’s antiviral function in shrimp, we conducted an RNAi experiment. As depicted in Figure 6A, LvMALT1 expression in the dsRNA-LvMALT1 group exhibited a significant reduction to approximately 0.17-fold compared to the dsRNA-GFP group during WSSV infection. Subsequently, we assessed the expression levels of PENs and CRUs in LvMALT1-silenced shrimp following WSSV challenge. Our results indicated a marked suppression of PENs and CRUs expression in dsRNA-LvMALT1-treated shrimp in comparison to their dsRNA-GFP-treated counterparts (Figure 6B,C). These findings strongly suggest that LvMALT1 can induce the expression of PENs and CRUs in WSSV-infected shrimp. Furthermore, we performed a comparative analysis of the mortality rate and viral loads in shrimp subjected to dsRNA-LvMALT1 and the dsRNA-GFP control. At 40 h post-WSSV challenge, shrimp from dsRNA-LvMALT1 group died faster than those of the dsRNA-GFP group (χ^2^: 9.35, *p* = 0.0022), and all shrimp had died by 104 h (Figure 6D). Additionally, elevated viral loads were observed at 48 h post-WSSV infection (Figure 6E), signifying that LvMALT1 knockdown rendered shrimp more susceptible to WSSV infection. These outcomes collectively suggest that LvMALT1 elicits an antiviral immune response through the NF-κB-AMPs pathway during WSSV infection.

## 4. Discussion

MALT1, predominantly located in vertebrate lymphocytes, plays a critical role in signal transduction from surface receptors such as the T-cell receptor (TCR) or the B-cell receptor (BCR) [39]. It serves as a vital component for the proper functioning of the NF-κβ pathway, which serves as a central regulator of gene expression controlling various facets of immune responses. Activated within the CBM complex, MALT1 recruits crucial downstream proteins, including TRAF6, to activate NF-κB-dependent gene expression [40].

In this study, we initially cloned and identified a MALT1 homolog, named LvMALT1, in the shrimp, *L. vannamei*. Our research focused on investigating the role of LvMALT1 within the shrimp’s NF-κβ pathway. LvMALT1 exhibited a structural configuration featuring a death domain at the N-terminus, two Ig-like domains, a putative caspase-like domain, and an Ig-like domain at the C-terminal, mirroring structural features found in other MALT1 homologs. Phylogenetic analysis revealed that LvMALT1 clustered with invertebrate MALT1 homologs, forming a distinct branch, indicating its membership within the invertebrate MALT1 family. To date, LvTRAF6 is the sole TRAF homolog associated with NF-κB activation in shrimp innate immunity [19]. The structural similarities between the domains of MALT1 and LvMALT1 led us to hypothesize that LvMALT1 may interact with LvTRAF6, potentially influencing NF-κB signaling. Indeed, our study discovered that LvMALT1 interacts with LvTRAF6. In mammals, MALT1’s ability to recruit TRAF6 and subsequently activate the IKK complex is attributed to two TRAF6 binding motifs situated outside the caspase-like domain [6]. Similarly, our findings showed that the C-terminal of LvMALT1 (301–787 aa) served as the binding region with LvTRAF6, akin to the MALT1 homologs in mammals.

Previous research has uncovered MALT1’s integral role in immunoreceptor-induced activation events, employing various strategies to facilitate NF-κB activation [38]. The *L. vannamei* NF-κB pathway is known to activate the expression of shrimp AMPs [19], but MALT1’s involvement in the shrimp NF-κB pathway remains insufficiently understood. To investigate LvMALT1’s function in NF-κB signal transduction, we conducted dual luciferase reporter assays. Our findings revealed that LvMALT1 and its truncated form, LvMALT1 (301–787 aa), significantly induced NF-κB pathway activation. Moreover, it appeared to function as an adapter protein by activating the promoters of *Drosophila* and shrimp AMP genes. To further explore LvMALT1’s role in immune response in vivo, we performed RNAi, which suggested that LvMALT1 might activate NF-κB-AMPs by enhancing the phosphorylation of LvDorsal and LvRelish.

Notably, there have been no reports thus far on the relationship between MALT1 homologs and virus infection or replication in crustaceans. Our study identified LvMALT1 as participating in immune responses to WSSV infection (Figure 3B). Further exploration of LvMALT1’s function during WSSV infection through RNAi experiments revealed its importance in defending against WSSV. In mammals, research has underscored the significance of MALT1 homologs in antiviral immune responses. MALT1 deficiency has been linked to impaired antiviral humoral immune responses. Notably, antiviral and inflammatory gene expression is significantly disrupted in the brain of MALT1^−/−^ mice at the pre-symptomatic phase of Evelyn–Rotnycki–Abelseth (ERA) virus infection, rendering it neurovirulent [41]. Furthermore, MALT1 has been shown to play an essential role in inhibiting HSV-1 replication [42]. Similarly, our findings indicate that LvMALT1 can counter viral infections through the activation of the NF-κB pathway.

It is worth noting that LvDorsal is primarily regulated by the Toll pathway, while LvRelish is mainly governed by the IMD pathway. Therefore, the activation of LvDorsal and LvRelish suggests the involvement of LvMALT1 in regulating the Toll–Dorsal pathway and the IMD–Relish pathway. Rapid and transient expression of AMPs has been deemed pivotal for shrimp’s defense against microbial infections [10]. Several AMPs regulated by the NF-κB pathway, including PENs and CRUs, have been shown to play significant roles in resisting WSSV infection. Conversely, the silencing of PENs or CRUs in shrimp led to higher viral burdens [18,43].

In summary, we have identified a MALT1 homolog in *L. vannamei* and explored its role during WSSV infection. Our results indicate that LvMALT1 can interact with LvTRAF6 and act as an intracellular signal transducer to activate NF-κB-mediated PENs and CRUs, thereby inhibiting WSSV replication. While further studies are needed to elucidate the molecular mechanisms underlying LvMALT1’s role in the activation of the NF-κB pathway, our findings provide substantial evidence that LvMALT1 is a potent effector of anti-WSSV activity and presents a promising target for resistant breeding in shrimp aquaculture disease control.

## Figures and Tables

**Table 1:** Primers used in this study.

Primer Name	Sequence (5′–3′)	Accession No.	Product Size (bp)
Protein expression			
MALT1-F	CGGGGTACCATGATGTCAACAGTAAAACCCAAG	XP_027207258.1	2361
MALT1-R	CCGCTCGAGCTAGTCAAGGCAATTCCAGTGTTC	XP_027207258.1	
MALT1(1–300)-F	CGGGGTACCATGATGTCAACAGTAAAACCCAAG	XP_027207258.1	300
MALT1(1–300)-R	CCGCTCGAGCTATGGAACATTAGTTACAATATTCTCAAG	XP_027207258.1	
MALT1(301–900)-F	CGGGGTACCATGGTGTCGGTTCTGAAGCCT	XP_027207258.1	600
MALT1(301–900)-R	CCGCTCGAGCTAATCGTCTAAACCATAAGAACTTTTC	XP_027207258.1	
MALT1(901–2361)-F	CGGGGTACCATGCGTGTTGACCTCTCCTTT	XP_027207258.1	1461
MALT1(901–2361)-R	CCGCTCGAGCTACTAGTCAAGGCAATTCCAGTG	XP_027207258.1	
dsRNA templates amplification			
LvMALT1-dsF	GTGAAAGTTGCTCCTCCT	XP_027207258.1	573
T7-LvMALT1-R	GGATCCTAATACGACTCACTATAGGAAACACGGCATAGTCTCC	XP_027207258.1	
T7-LvMALT1-F	GGATCCTAATACGACTCACTATAGGGTGAAAGTTGCTCCTCCT	XP_027207258.1	573
LvMALT1-dsR	AAACACGGCATAGTCTCC	XP_027207258.1	
dsGFP-F	TTGAAGTTCACCTTGATGCC	DQ389577	529
dsGFP-T7-R	GGATCCTAATACGACTCACTATAGGTTGAAGTTCACCTTGATGCC	DQ389577	
dsGFP-T7-F	GGATCCTAATACGACTCACTATAGGATGGTGAGCAAGGGCGAGGA	DQ389577	529
dsGFP-R	TTGAAGTTCACCTTGATGCC	DQ389577	
Quantitative RT-PCR			
MALT1-qF	TATGGAACGGATGAGGGT	XP_027207258.1	201
MALT1-qR	TTCTTGAGGCTTTGGTGG	XP_027207258.1	
LvEF-1α-F	TATGCTCCTTTTGGACGTTTTGC	GU136229	118
LvEF-1α-R	CCTTTTCTGCGGCCTTGGTAG	GU136229	
LvPEN2-qF	GACGGAGAAGACAATGGAAACC	DQ206401.1	160
LvPEN2-qR	ATCTTTAGCGATGGATAGACGAA	DQ206401.1	
LvCRU1-qR	GTAGGTGTTGGTGGTGGTTTC	XM_027352254.1	174
LvCRU1-qR	CTCGCAGCAGTAGGCTTGAC	XM_027352254.1	
LvPEN3-qF	TACAACGGTTGCCCTGTCTCA	XM_027360479.1	105
LvPEN3-qR	ACCGGAATATCCCTTTCCCAC	XM_027360479.1	
LvCRU2-qF	GGTACGTCTGCTGCAAGCC	XM_027368306.1	173
LvCRU2-qR	CTGAGAACCTGCCACGATGG	XM_027368306.1	
LvPEN4-qF	GGTGCGATGTATGCTACGGAA	DQ211701.1	106
LvPEN4-qR	CATCGTCTTCTCCATCAACCA	DQ211701.1	
LvCRU3-qF	TCCACAATGGTCAGCGTCAAG	MT375586.1	197
LvCRU3-qR	CTGTCCGACAAGCAGTTCCTC	MT375586.1	

## Data Availability

All reagents and experimental data are available within Transparent Methods or from corresponding author upon reasonable request.
